# Will the Scottish Cancer Target for the year 2000 be met? The use of cancer registration and death records to predict future cancer incidence and mortality in Scotland.

**DOI:** 10.1038/bjc.1996.214

**Published:** 1996-05

**Authors:** L. Sharp, R. J. Black, C. S. Muir, I. Gemmell, A. R. Finlayson, E. F. Harkness

**Affiliations:** Scottish Cancer Intelligence Unit, Information & Statistics Division, National Health Service in Scotland, Edinburgh, UK.

## Abstract

Cancer mortality data reflect disease incidence and the effectiveness of treatment. Incidence data, however, reflect the burden of disease in the population and indicate the need for prevention measures, diagnostic services and cancer treatment facilities. Monitoring of targets mandates that both be considered. The Scottish Cancer Target, established in 1991, proposed that a reduction of 15% in mortality from cancer in the under-65s should be achieved between 1986 and 2000. Each year in Scotland approximately 8300 persons under 65 are diagnosed with cancer and 4500 die from the disease. The most common malignancies, in terms of both incident cases and deaths, in the under-65s, are lung and large bowel cancer in males, and breast, large bowel and lung cancer in females. A decrease of 6% in the number of cancer cases diagnosed in males under 65 is predicted between 1986 and 2000, whereas the number of cases in females in the year 2000 is expected to remain at the 1986 level. In contrast, substantial reductions in mortality are expected for both sexes: 17% and 25% in males and females respectively. Demographic changes will influence the numbers of cancer cases and deaths in the Scottish population in the year 2000. However, long-term trends in the major risk factors, such as smoking, are likely to be the most important determinants of the future cancer burden.


British Journal of Cancer (1996) 73, 1115-1121
© 1996 Stockton Press All rights reserved 0007-0920/96 $12.00

### Will the Scottish Cancer Target for the year 2000 be met? The use of cancer registration and death records to predict future cancer incidence and mortality in Scotland

L Sharp[1], RJ Black[1,2], CS Muir[1], I Gemmell[1], AR Finlayson[1] and EF Harkness[1]

[1]*Scottish Cancer Intelligence Unit, Information & Statistics Division, National Health Service in Scotland, Trinity Park House, South Trinity Road, Edinburgh EH5 3SQ, UK;* [2]*Unit of Descriptive Epidemiology, International Agency for Research on Cancer, 150 Cours Albert Thomas, 69372 Lyon Cedex 08, France.*

**Summary** Cancer mortality data reflect disease incidence and the effectiveness of treatment. Incidence data, however, reflect the burden of disease in the population and indicate the need for prevention measures, diagnostic services and cancer treatment facilities. Monitoring of targets mandates that both be considered. The Scottish Cancer Target, established in 1991, proposed that a reduction of 15% in mortality from cancer in the under-65s should be achieved between 1986 and 2000. Each year in Scotland approximately 8300 persons under 65 are diagnosed with cancer and 4500 die from the disease. The most common malignancies, in terms of both incident cases and deaths, in the under-65s, are lung and large bowel cancer in males, and breast, large bowel and lung cancer in females. A decrease of 6% in the number of cancer cases diagnosed in males under 65 is predicted between 1986 and 2000, whereas the number of cases in females in the year 2000 is expected to remain at the 1986 level. In contrast, substantial reductions in mortality are expected for both sexes: 17% and 25% in males and females respectively. Demographic changes will influence the numbers of cancer cases and deaths in the Scottish population in the year 2000. However, long-term trends in the major risk factors, such as smoking, are likely to be the most important determinants of the future cancer burden.

**Keywords:** cancer; incidence; mortality; targets; projections; Scotland

The Scottish health targets were established in 1991 in *Health Education in Scotland–A National Policy Statement* (SHHD, 1991) and reiterated in *Scotland's Health–A Challenge to Us All* (SHHD, 1992). Among targets pertaining to the major causes of morbidity and mortality in the Scottish population was one directed specifically towards cancer: 'to reduce mortality from cancers in the under-65s by 15% between 1986 and 2000', that is, a reduction from 104 deaths per 100 000 to 88 deaths per 100 000 by the year 2000.

Since the last century information on cause of death has been recorded routinely by the Registrar General for Scotland and used to monitor trends in mortality from cancer. However, to assess the burden of disease it is necessary to be able to measure cases newly diagnosed in the population. Scotland is fortunate in that it is one of the few countries in the world with long-standing, national, population-based cancer registration. The Scottish National Cancer Registration Scheme aims to collect information on all incident cases of cancer in Scotland and has a database extending back to 1958. The availability of these long series of data provides an opportunity to estimate cancer incidence and mortality in future years. The purpose of this paper is to facilitate the assessment of progress towards attaining the Scottish Cancer Target by describing trends in incidence of, and mortality from, the common cancers over recent decades and presenting projected numbers of cases and deaths in the year 2000.

### Data and methods

Registrations of cancer (ICD-9 140–208) in the period 1968–92 were extracted from the national cancer database held by the Information & Statistics Division of the National Health Service in Scotland. Non-melanoma skin cancer (ICD-9 173) registrations were excluded as they are unlikely to be complete. With the permission of the General Register Office (Scotland), individual mortality records for 1968–92 with cancer recorded as the primary cause of death were extracted from computerised death listings.

A statistical model based on birth cohort, age and time period (Robertson and Boyle, 1986) was used to estimate the projected number of cancer registrations and deaths in Scotland in the year 2000. This method partially addresses the problem of overlapping birth cohorts by using individual cancer registration and mortality records to assign each individual to a unique cohort. Records were tabulated by period of diagnosis (or death) (1968–72, 1973–77, ..., 1988–92), age at diagnosis (or death) (25–29, 30–34, ..., 80–84) and birth cohort (1883–87, 1888–92, ..., 1963–67). For each cancer site included in the analysis, a log-linear model was fitted in GLIM (NAG, 1985), with adjustment for extra-Poisson variation where necessary (Breslow, 1984), to generate estimates of age, time period and birth cohort effects for registration and mortality data separately. The age effects represent differing risks associated with different age groups and may reflect cumulative exposure to cancer causing agents; the time period effects describe changes in rates occurring in all age groups simultaneously and may reflect, for example, increasing completeness of registration or the introduction of a new diagnostic technique; the birth cohort effects represent changes in rates in successive generations and may reflect differences in lifestyle or cultural habits such as reproductive practices or tobacco or alcohol consumption. Only those sites of cancer for which at least 200 cases were diagnosed in the age group 0–64 years in 1986 were included in these analyses. Males and females were considered separately. The individual cancers analysed were stomach (ICD-9 151), large bowel (ICD-9 153+154), lung (ICD-9 162), and bladder (ICD-9 188) in males and large bowel (ICD-9 153+154), lung (ICD-9 162), breast (ICD-9 174), cervix uteri (ICD-9 180) and ovary (ICD-9 183) in females. All malignant neoplasms (ICD-9 140–208, excluding ICD-9 173) were also considered as a single category.

It was assumed that the cancer registration and death rates in the 0–24 age group in the period 1981–90 (Sharp *et al.*, 1993: Registrar General, 1981–90) would persist to the year 2000 and that the period effects for 1988–92 from the fitted

Correspondence: L Sharp, Department of Medicine & Therapeutics, University of Aberdeen, Polwarth Building, Foresterhill, Aberdeen AB9 2ZD, UK
Received 19 May 1995; revised 30 October 1995; accepted 31 October 1995

models would continue in future periods. Estimates of age and birth cohort specific incidence and mortality rates from the fitted models were applied to population projections (Registrar General, 1993) to produce projected numbers of cases and deaths in the 0–64 age group. Percentage changes between the model estimates for 1986 and 2000 were calculated and applied to the actual 1986 registrations and deaths to obtain projected numbers in the year 2000.

The results are presented in two sections. The first section concerns cancer in Scotland in the baseline year 1986. Numbers and percentages of the most common cancers in the under 65 population are presented. Five year relative survival rates were calculated as described by Black and co-workers (1993) for patients aged under 65 and diagnosed in two periods, 1968–72 and 1983–87. Incidence and mortality rates for all malignant neoplasms in the under-65s in 1986, directly standardised with respect to the world population (Segi, 1960), were obtained from the EUROCIM package (European Network of Cancer Registries, 1995) for Scotland and selected European cancer registries. The second section examines time trends in incidence and mortality and presents the projected numbers of registrations and deaths in the year 2000. Both crude and age-standardised rates are used depending on circumstances. To facilitate comparison with the Scottish Cancer Target, which is expressed in terms of unadjusted rates, crude incidence and mortality rates for individual cancer sites in 1986 and 1992 are shown. To permit assessment of long-term time trends in incidence and mortality, age-standardised rates (to the world population) for the period 1960–1992 were derived from Black et al. (1995). These data are presented for all malignant neoplasms combined as well as for individual cancer sites.

### Results

*Cancer in Scotland in the baseline year 1986*

*Incidence* In 1986, 10 795 males and 11 109 females of all ages were registered with cancer (non-melanoma skin cancer excluded) in Scotland. Of this total, 38% (8313) of cases occurred in those aged under 65 years, 56% (12 190) in the 65 to 84 age group and the remaining 6% (1401) in those aged 85 and older. The proportion of cancers presenting before 65 years in females (40%: 4452 registrations) was greater than that in males (36%: 3861) due, in the main, to cancer of the reproductive system in females.

The numbers and percentages of registrations for the most frequent cancers in persons under 65 years of age in 1986 are shown in Table I. In males, cancer of the lung accounted for the greatest proportion of registrations (28%), followed by cancers of the large bowel (11%) and bladder (7%). In females, 31% of registrations were due to breast malignancies with a further 14% due to cancers of the cervix and ovary. Cancers of the lung and large bowel were also common (12% and 9% respectively).

*Mortality* Almost a third (4539) of all cancer deaths occurred in those aged under 65 years, a further 60% (8758) in the 65–84 age group and 8% (1194) in those aged 85 and older. Lung tumours accounted for 40% of cancer deaths in males and 20% in females (Table I). In total 41% of deaths in females under 65 were ascribed to cancers of the reproductive system.

*Survival* Relative survival rates at five years after diagnosis for all cancer patients under 65 years diagnosed in 1983–87

**Table II** Five year relative survival rates (%) for persons aged under 65 at diagnosis, by site of cancer, sex and period of diagnosis, 1968–72 and 1983–87

| | Period of diagnosis | |
|---|---|---|
| *Site of cancer* | *1968–72* | *1983–87* |
| *Males* | | |
| Stomach (ICD-9 151) | 7.9 | 12.2 |
| Large bowel (ICD-9 153+154) | 34.1 | 41.0 |
| Lung (ICD-9 162) | 10.1 | 8.6 |
| Bladder (ICD-9 188) | 63.9 | 76.3 |
| All malignant neoplasms (ICD-9 140–208)[a] | 25.0 | 33.0 |
| *Females* | | |
| Large bowel (ICD-9 153+154) | 37.3 | 42.3 |
| Lung (ICD-9 162) | 8.7 | 9.0 |
| Breast (ICD-9 174) | 56.8 | 66.3 |
| Cervix (ICD-9 180) | 59.6 | 64.2 |
| Ovary (ICD-9 183) | 28.9 | 37.9 |
| All malignant neoplasms (ICD-9 140–208)[a] | 42.9 | 49.4 |

[a]Excludes non-melanoma skin cancer (ICD-9 173).

**Table I** Numbers and percentages of registrations of, and deaths from, the most common cancers in persons aged under 65, by sex and site of cancer, 1986

| *Site of cancer* | *No. of registrations* | *%* | *No. of deaths* | *%* |
|---|---|---|---|---|
| *Males* | | | | |
| Lung (ICD-9 162) | 1064 | 27.6 | 941 | 40.2 |
| Large bowel (ICD-9 153+154) | 437 | 11.3 | 211 | 9.0 |
| Bladder (ICD-9 188) | 277 | 7.2 | | |
| Stomach (ICD-9 151) | 229 | 5.9 | 137 | 5.9 |
| Testis (ICD-9 186) | 150 | 3.9 | | |
| Oesophagus ICD-9 150) | | | 125 | 5.3 |
| Brain and central nervous system (ICD-9 191+192) | | | 97 | 4.1 |
| Other sites | 1704 | 44.1 | 830 | 35.5 |
| All malignant neoplasms (ICD-9 140–208)[a] | 3861 | 100.0 | 2341 | 100.0 |
| *Females* | | | | |
| Breast (ICD-9 174) | 1366 | 30.7 | 571 | 26.0 |
| Lung (ICD-9 162) | 527 | 11.8 | 452 | 20.6 |
| Large bowel (ICD-9 153+154) | 389 | 8.7 | 196 | 8.9 |
| Cervix (ICD-9 180) | 334 | 7.5 | 105 | 4.8 |
| Ovary (ICD-9 183) | 269 | 6.0 | 157 | 7.1 |
| Other sites | 1567 | 35.2 | 717 | 32.6 |
| All malignant neoplasms (ICD-9 140–208)[a] | 4452 | 100.0 | 2198 | 100.0 |

[a]Excludes non-melanoma skin cancer (ICD-9 173).

was 33% for males and 49% for females (Table II). With the exception of lung cancer, survival from most common cancers improved between 1968–72 and 1983–87 (Table II).

*International comparisons* Table III shows age-standardised incidence and mortality rates for all malignant neoplasms in Scotland and selected European cancer registries in 1986. Incidence and mortality in both sexes in Scotland belonged towards the top of the range of rates reported across Europe. The incidence of malignant neoplasms in Scottish males and females aged under 65 exceeded that in England and Wales by some 19% and 10% respectively. There were smaller differentials between the countries in mortality; rates were 15% and 6% higher in Scottish males and females respectively.

*Trends in the common cancers and projected registrations and deaths*

*All malignant neoplasms (ICD-9 140–208, excluding 173)* Since 1960 there have been steady increases in the age-standardised incidence of all malignant neoplasms in persons of both sexes aged under 65 in Scotland (Figure 1). Rates in males rose by 17% from 122 per 100 000 in 1960 to 143 per 100 000 in 1992 and those in females by 43% from 120 to 172. For both sexes, the most substantial increases in incidence were observed in those aged 55–64 years at diagnosis (data not shown). Over the same time period, cancer mortality in males declined consistently, from 99 to 77 per 100 000, whereas mortality in females remained constant at around 75 per 100 000.

**Table III** Age-standardised[a] incidence and mortality rates (per 100000), all malignant neoplasms[b], 0–64 years, Scotland and selected European cancer registries, 1986

| | *Incidence* | | *Mortality* | |
|---|---|---|---|---|
| *Country and registry* | *Males* | *Females* | *Males* | *Females* |
| Scotland | 141.5 | 155.7 | 84.4 | 73.5 |
| Denmark | 131.8 | 171.1 | 76.2 | 77.7 |
| Netherlands: south | 131.3 | 145.8 | 76.8 | 63.6 |
| Spain: Tarragona | 118.7 | 116.5 | 73.1 | 51.8 |
| England and Wales | 118.7 | 141.8 | 73.5 | 69.3 |
| Poland: Cracow | 118.6 | 119.8 | 90.5 | 49.1 |

[a]World standard population. [b]Excludes non-melanoma skin cancer (ICD-9 173).

**Figure 2** Crude incidence rates (per 100000) by site of cancer, sex and year of diagnosis, 0–64 years, 1986 (■) and 1992 (□).

**Figure 1** Age-standardised* incidence (◇, males; ○, females) and mortality rates (◆, males; ●, females) (per 100000), by year and sex, all malignant neoplasms, 0–64 years, 1960–92. *World standard population.

**Figure 3** Crude mortality rates (per 100000) by site of cancer, sex and year of death, 0–64 years, 1986 (■) and 1992 (□).

**Table IV** Actual and projected numbers of registrations and estimated percentage changes from 1986 to 2000, for persons aged under 65 at diagnosis, by site of cancer and sex

| | *No. of registrations 1986* | | *Projected registrations 2000* | | *Estimated % change* | |
|---|---|---|---|---|---|---|
| *Site of cancer* | *Males* | *Females* | *Males* | *Females* | *Males* | *Females* |
| Stomach (ICD-9 151) | 229 | | 161 | | −30 | |
| Large bowel (ICD-9 153+154) | 437 | 389 | 441 | 333 | 1 | −15 |
| Lung (ICD-9 162) | 1064 | 527 | 650 | 362 | −39 | −31 |
| Breast (ICD-9 174) | | 1366 | | 1518 | | 11 |
| Cervix (ICD-9 180) | | 334 | | 428 | | 28 |
| Ovary (ICD-9 183) | | 269 | | 255 | | −5 |
| Bladder (ICD-9 188) | 277 | | 255 | | −8 | |
| All malignant neoplasms (ICD-9 140–208)[a] | 3861 | 4452 | 3641 | 4461 | −6 | 0 |

[a]Excludes non-melanoma skin cancer (ICD-9 173).

Table V Actual and projected numbers of deaths and estimated percentage changes from 1986 to 2000, for persons aged under 65 at death, by site of cancer and sex

| Site of cancer | No. of deaths 1986 | | Projected deaths 2000 | | Estimated % change | |
|---|---|---|---|---|---|---|
| | Males | Females | Males | Females | Males | Females |
| Stomach (ICD-9 151) | 137 | | 84 | | −39 | |
| Large bowel (ICD-9 153+154) | 211 | 196 | 188 | 139 | −11 | −29 |
| Lung (ICD-9 162) | 941 | 452 | 583 | 258 | −38 | −43 |
| Breast (ICD-9 174) | | 571 | | 451 | | −21 |
| Cervix (ICD-9 180) | | 105 | | 107 | | 2 |
| Ovary (ICD-9 183) | | 157 | | 105 | | −33 |
| Bladder (ICD-9 188) | 52 | | 47 | | −10 | |
| All malignant neoplasms (ICD-9 140–208)[a] | 2341 | 2198 | 1943 | 1649 | −17 | −25 |

[a]Excludes non-melanoma skin cancer (ICD-9 173).

In 1986 the crude incidence rates of all cancers in those under 65 were 176 per 100 000 in males and 204 per 100 000 in females (Figure 2). The rate for males was little changed by 1992, whereas incidence in women had risen by 11%. Mortality in 1986 was 107 and 101 per 100 000 for males and females respectively (Figure 3). By 1992 this had fallen by 8% in males and 3% in females.

In comparison with the baseline year, 1986, the number of cancers diagnosed in those under 65 years in Scotland in 2000 is forecast to fall by 6% to 3641 in males and increase marginally (0.2%) to 4461 in females (Table IV). Substantial decreases in deaths are projected with the total in males falling by 17%, from 2341 to 1943, and in females by 25%, from 2198 to 1649 (Table V).

*Cancer of the stomach (ICD-9 151)* Incidence and mortality rates for stomach cancer in males under 65 have declined steadily over the last three decades. In 1986, approximately one-third of the incident cases and a quarter of deaths in males occurred in those under 65. Crude mortality in 1992 (5 per 100 000) was 18% lower than in the baseline year, 1986. In the years to 2000 the decrease in stomach cancer is expected to continue; the projected number of registrations represents a decrease of 30% on the 1986 figure. A fall of a similar magnitude in the number of deaths is expected (39%) with rates decreasing in all but the youngest age groups (data not shown).

*Cancer of the large bowel (ICD-9 153+154)* Incidence data for large bowel cancer in those under 65 indicate a small, but steady, increase over the past 30 years. Mortality has shown a smaller, consistent, downward trend. For both sexes the crude incidence and death rates changed little between 1986 and 1992. The projected number of cases of large bowel cancer in persons under 65 in the year 2000 is 774, comprising a rise of 1% in males and a fall of 15% in females on the 1986 figures. For both sexes, substantial decreases in mortality are anticipated; 11% in males and 29% in females. Relative survival has improved over the past 15 years for both sexes with a proportionately greater rise in males (Table II).

*Cancer of the trachea, bronchus and lung (ICD-9 162)* In 1986, in those aged under 65, there were 1591 registrations of, and 1393 deaths from, lung cancer. This accounted for 19% of all new cancers and 33% of cancer deaths. Mortality rates for lung cancer in Scottish males under 65 have been declining since the mid 1960s with incidence falling since 1980. Between 1986 and 1992 crude incidence and mortality rates fell by 17% and 20% respectively. For females under 65, both age-standardised incidence and mortality rates have shown a steady upward trend since 1960. The number of incident cases in 1992 slightly exceeded that in 1986. Decreases of 39% and 31% in the numbers of cases diagnosed in males and females respectively are expected between 1986 and 2000. Falls of a similar magnitude are predicted from mortality data (38% and 43%) with the greatest reductions expected to occur in the younger age groups.

*Cancer of the female breast (ICD-9 174)* A significant proportion of breast tumours develop in women aged under 65 (52%) and 44% of deaths occur in this age group. Incidence of breast cancer has risen steadily since 1960, with the number of incident cases diagnosed increasing substantially between 1986 and 1992. Little changed has been observed in mortality. In the year 2000, 1518 registrations are projected, an 11% increase on the number in the baseline year. The rise comprises, in the main, an increase in the numbers of tumours in women aged 50–64. It is predicted that the numbers of deaths due to cancer of the breast in those under 65 will fall by 21% between 1986 and 2000.

*Cancer of the cervix uteri (ICD-9 180)* The majority of cervical neoplasia are diagnosed in women aged less than 65 years (72%) and more than one-half of deaths occur in this age group. A regular downward trend in age-standardised mortality rates is apparent since the 1960s. There is no consistent pattern in incidence over the past 30 years; rates were lower in the 1970s than the previous decade but rose from 1980 to 1990. By 2000, a substantial increase in the numbers of invasive cancers of the cervix diagnosed in the under 65s is forecast; 28% higher than the 1986 figure. Numbers of deaths are predicted to be little changed from the 1986 total.

*Cancer of the ovary (ICD-9 183)* Ovarian cancer incidence and mortality rates in the under-65s have diverged over the last 15–20 years. While incidence has increased since 1960, mortality has been falling since the mid-1970s. Around half of ovarian malignancies and 40% of deaths occur in women aged under 65. Both crude incidence and mortality rates were similar in 1992 to the baseline year 1986. A small (5%) decrease in the number of incident cases in the year 2000 is forecast. However, a substantial decrease (33%) in numbers of deaths is predicted with falls of the greatest magnitude in younger women.

*Cancer of the bladder (ICD-9 188)* Approximately a third of male bladder cancer cases are diagnosed in the under-65s, but, as relative survival at five years after diagnosis exceeds 75%, only 17% of deaths occur in this age group. Standardised registration rates for males have shown a steady increase over the past 30 years although mortality has remained fairly constant. There was little difference in incidence or mortality rates between 1986 and 1992. Falls of similar magnitude in both incidence and mortality are expected in the year 2000; 8% and 10% decreases in cases and deaths respectively.

### Discussion

### *Predicted mortality and incidence in the year 2000 in those under 65 years*

A substantial reduction in the numbers of deaths due to cancer in the population aged under 65 in Scotland is predicted between 1986 and 2000. A total of 3592 cancer deaths in those under 65 is expected in the year 2000, deaths in those under 65 is expected in the year 2000, representing decreases of 17% (from 2341 in 1986 to 1943 in 2000) and 25% (2198 to 1649) in males and females respectively. On the basis of these estimates, the Scottish Cancer Target, of a 15% reduction in mortality in the under 65s between 1986 and 2000 (SHHD, 1991), will be attained. In contrast, over the same time period, a 3% decrease in the number of newly diagnosed cancer cases is forecast, comprising a fall of 6% in males but little change in females. In 2000, in those aged less than 65 years, a total of 8102 incident cancer cases are predicted, 55% of these occurring in women.

Data for all malignant neoplasms are difficult to interpret and may conceal divergent patterns for specific cancers. In men, substantial falls in the numbers of cases of, and deaths from, stomach and lung cancer can be expected. In women under 65, a reduction in the number of lung neoplasms is also anticipated. This is discussed more fully below. Women are also expected to experience increases in the numbers of diagnosed tumours of the cervix and, to a lesser extent, breast. In contrast, mortality is predicted to remain unchanged for cervical cancer and to decline for breast cancer. Organised screening exists for these cancers (Warner *et al*., 1993; Strong, 1986) and is expected to influence trends in both incidence and mortality greatly.

The specific cancer sites examined in this paper account for around 60% of diagnosed cases and deaths from all malignant neoplasms in persons under 65 years in Scotland. Over the 10 years from 1981 to 1990 the incidence rates of some other cancers not examined in this paper have risen significantly. Malignant melanoma, non-Hodgkin's lymphoma and testicular cancer have shown the greatest increases in incidence (Sharp *et al*., 1993). For example, between 1981 and 1990 the numbers of malignant melanoma cases diagnosed in the under-65s rose from 156 to 325 and the numbers of non-Hodgkin's lymphomas doubled from 73 to 148. Substantial proportions of these cancers are diagnosed in those aged less than 65. In women, in particular, incidence rates of oesophageal and larynx cancer are also rising, albeit to a lesser extent, but these cancers constitute only a small proportion of all neoplasms in those under 65 years. In common with trends observed in other countries, the incidence of tumours of the prostate in Scottish men has been rising and is expected to continue to rise: around 14% of cases are diagnosed in those aged under 65.

*Features of the statistical model*

The model used to generate the projected numbers of cases and deaths incorporates the combined effects of age at diagnosis (or death), period of diagnosis (or death) and birth cohort. It is well established that such a model is likely to produce a more accurate description of time trends in disease than one based simply on linear extrapolation (Clayton and Schifflers, 1987).

On occasion the trends predicted for incidence and mortality may seem discrepant – as for large bowel cancer. Such an event may reflect not only changes in the time-lag between diagnosis and death but also birth cohort-specific improvements in survival associated perhaps with earlier stage tumours in recent generations. The observations for large bowel cancer are not unique to Scotland. In the US, mortality from colon cancer is rising significantly in black males, with little change in black women and a fall in whites of both sexes. Incidence rose significantly in both black and white men and in black females between 1973 and 1990 yet there was a significant drop for white men and women from 1986 to 1990 (Miller *et al*., 1993). Under such circumstances age–period–cohort models are likely to describe the underlying trends more accurately than cross-sectional data.

The estimates of the age, period and birth cohort effects generated by the model are subject to errors which are, of course, proportionately greatest for less frequent cancers. Therefore, the site-specific estimates of percentage change in incidence will be more robust statistically than those for mortality. Also, the projections for the most common cancers (e.g. male lung cancer, female breast cancer) are more reliable than those for other sites.

The introduction of new diagnostic techniques or advances in therapy for cancer may be assumed to represent period effects if they occur in all age groups simultaneously. However, it is difficult to predict future developments in these areas and the model assumes that the period effect in 1988–92 will continue unchanged to 2000. Further, it is unlikely that new diagnostic methods or treatments are, or will be, introduced at the same time and to the same extent in all age groups. For example, the Scottish Breast Screening Programme (SBSP) offers mammography to women aged 50–64 years (Warner *et al*., 1993). Although the long-term aim of the screening programme is to reduce mortality by 25% in the screened age group by the year 2000, in the short term incidence should rise. The magnitude of this rise is difficult to quantify and has not been incorporated in the statistical model. Therefore, it is likely that the incidence model will over-estimate to some extent the number of new cases of breast cancer in 2000. However, since the SBSP achieved national coverage in 1991, the mortality model describes the trends likely to occur in the absence of systematic screening. Thus, the prediction of a decrease of 21% in the number of deaths due to cancer of the breast in women under 65 between 1986 and 2000 may under-estimate the actual reduction achieved should screening prove effective. The combined effect of long-term trends in mortality and the introduction of national breast screening cannot be quantified as it is, as yet, too early to formally evaluate the screening programme.

Cytological screening for cervical cancer, aimed at detecting lesions in the preclinical phase, has been offered on a systematic basis in some parts of Scotland for more than 30 years (Macgregor *et al*., 1994). Screening policies have varied across the country and over time. Consequently, the effects of cervical screening on the statistical models for both incidence and mortality are likely to be complex and cannot be adequately assessed.

*Trends in cancer risk factors*

For many of the cancers diagnosed in Scotland the risk factors are well understood. Muir (1993) estimates that at least one-third of all tumours in males and 17% in females are probably caused by smoking and a further 6% and 3% caused by the combined effects of tobacco and alcohol. As tobacco consumption, in particular, is implicated in the aetiology of many of the cancers common in Scotland, past and current patterns of tobacco use in the population will dictate a large part of future cancer incidence and mortality trends. From 1960 to 1980 the incidence of cancer of the lung rose steadily in Scottish men; since that time both incidence and mortality rates have been declining. For women a consistent upward trend was still apparent into the 1990s (ISD, 1994). These data reflect the very strong birth cohort effect in lung cancer (Black *et al*., 1995) related to patterns of smoking behaviour in successive generations. For men the peak incidence occurred when the birth cohorts and age groups of highest risk coincided at the end of the 1970s. As smoking became prevalent among women some years later than in men, it is predicted that incidence will not peak until around 2000. Data on tobacco use in Scotland (ISD, 1994) reveal that the decline in smoking prevalence in women, notably younger women, has been less pronounced than in men. This may mean that the future decreases in lung cancer incidence and mortality in younger women will not be of the magnitude forecast in this paper.

The proportions of neoplasms in the Scottish population likely to be caused by aspects of lifestyle, including diet and reproductive factors, have been estimated to be 32% in males and 61% in females (Muir, 1993). Diet now appears to be important in the genesis of several common cancers, although the contribution of diet to total cancer incidence and mortality cannot be quantified on the basis of present knowledge (WHO, 1990). The Scottish diet is typically low in fruit, vegetable and fibre consumption and high in saturated fat, refined sugar and salt intake (SHHD, 1993). Given that dietary patterns are often established at a very early age, it is likely that efforts to improve diet will be slow to take effect.

The frequencies of cancers of the breast, ovary and endometrium are influenced by past trends in reproductive factors in the population, particularly those associated with ovarian activity. Increased risk of breast cancer is established in relation to early age at menarche, late menopause, late age at first full-term pregnancy and low parity. The last of these is also an important risk factor for ovarian and endometrial cancer (Higginson *et al.*, 1992). Exogenous hormones also have a role in the aetiology of female cancers. While there is conclusive evidence that use of combined oral contraceptives protects against tumours of the uterus and ovary (Tomatis, 1990), studies have suggested that their use in adolescent women may result in a slight increase in risk of breast cancer (UK National Case-Control Study Group, 1989; Peto, 1989). In Scotland, the birth rate declined from 88.0 per 1000 women aged 15–44 in 1975 to 57.8 per 1000 in 1993 and the mean age at first pregnancy continues to rise (ISD, 1994). Data from the Family Planning (Clinic) Services (ISD, 1994) indicate that, in 1993, almost 50% fewer women using this service chose oral contraceptives as a form of contraception than in 1975. In the main, however, population-based data on trends in most reproductive factors is either unavailable or limited. Furthermore, the effects of such factors are likely to be synergistic. It is therefore difficult to estimate the overall impact that changes in these factors may have on the future burden of cancer in Scotland.

### *Demographic change*

Cancer is predominantly a disease of the elderly and the majority of incident cases and deaths occur in persons older than 65. It is projected, from the age–period–cohort analyses, that the number of cancers diagnosed in those aged 65–84 years will rise by 6% between 1986 and 2000, compared with a fall of 3% for those aged under 65. A sizeable increase (20%) in breast cancer cases in women in the 65–84 age group (who are not routinely invited for screening) is predicted, with a less than 10% reduction in numbers of deaths. In addition, a large rise in numbers of lung tumours diagnosed in women aged 65–84 is projected (31%); the overall number of lung cancers in women aged up to 84 years is forecast to rise by 8% between 1986 and 2000.

Demographic changes are likely to be an important determinant of the future cancer burden. Growth in the population at risk of the disease or changes in the population structure (e.g. increasing proportions of older persons at higher risk of developing or dying from cancer) may result in increases in the number of cases or deaths even if the age-specific risks do not alter, or indeed, fall. Between 1991 and 2001 increases of 15% and 12% in the population aged 30–64 in Scotland are expected for males and females respectively (Registrar General, 1993) with even greater growth will be greatest in the numbers of very elderly. The population aged 85 and older is forecast to double. Cancer is likely to become a public health concern of increasing dimensions in this age group.

### *Complementary nature of incidence and mortality data.*

Several authors have reviewed the relative value of cancer incidence and mortality data (e.g. Barker, 1984; Boyle, 1989). The routine availability of mortality data over many years has meant that mortality rates have become the most commonly used indicator of trends in cancer in human populations. However, mortality rates are the product of trends in both incidence of, and survival from, cancer. Therefore, as survival for some cancers improves, the use of mortality data in health monitoring becomes increasingly inappropriate. This happens in two ways. First, greater proportions of cancer patients who are treated successfully and die of another cause will result in a fall in mortality but not in the numbers of new patients seeking treatment and using health care resources. Second, longer survival times mean that mortality data reflect incidence of the disease with an increasing delay which makes trends difficult to interpret. Incidence data are the appropriate indicators for predicting future needs for prevention and early detection measures, diagnostic services and cancer treatment facilities.

### *Primary prevention of cancer mortality*

Mortality from cancer in a population may be influenced by primary, secondary and tertiary prevention. Several of the most common cancers are the result of many years of cumulated exposure to carcinogens such as tobacco and alcohol and less well understood risk factors such as lack of intake of fresh fruit and vegetables (Higginson *et al.*, 1992). Primary prevention, instituted through programmes of health promotion for example, is unlikely to effect an immediate reduction in cancer mortality and represents a long-term investment in improving the health of the population.

### *Role of earlier diagnosis and therapy*

For some cancers prognosis is directly related to the stage of the disease at diagnosis (Ponten, 1991). Hence, mortality may be influenced by detecting tumours earlier in their natural history. Secondary prevention aims to promote early diagnosis of disease, through, for example, systematic screening programmes, health education campaigns, or increased public awareness of cancer. Such initiatives, however, may take some time to influence survival and mortality rates. The thickness of malignant melanomas diagnosed in the west of Scotland was significantly reduced following a campaign promoting earlier reporting (MacKie and Hole, 1992). However, since five year survival for malignant melanoma for patients diagnosed in the mid-1980s exceeded 70% (Black *et al.*, 1993), it is likely to be some time before mortality is affected. Similarly, the reduction in mortality from cancer of the breast following the introduction of mammographic screening in Sweden took several years to become apparent (Tabar *et al.*, 1985). Improved diagnostic techniques may also result in earlier diagnosis but such advances are difficult to anticipate.

The introduction of effective therapy is the route most likely to produce immediate and substantial reductions in mortality from cancer. For example, in the mid-1970s when combination chemotherapy utilising cisplatin was introduced for the treatment of testicular cancer, mortality fell sharply (Boyle *et al.*, 1987). To date however, increased survival as a direct result of advances in treatment has only been solidly established for testicular tumours, some childhood and young adult cancers (Birch *et al.*, 1988; Hawkins, 1989) and Hodgkin's disease (Boyle *et al.*, 1988). Further developments in treatment for the more common cancers are difficult to predict. However, survival and, by extension, mortality may also be influenced by the organisation and availability of cancer diagnosis and treatment facilities. Studies have suggested that patients treated at specialist centres or teaching hospitals may have improved prognosis (Stiller, 1994; Harding *et al.*, 1993; Gillis *et al.*, 1991; Karjalainen, 1990). The use of treatment protocols, both within and outwith clinical trials (Karjalainen and Palva, 1989) and treatment by a specialist (Junor *et al.*, 1994; McArdle and Hole, 1991) have been shown to increase survival. Increasing the proportion of patients offered the best available therapy is likely to generate improvements in survival and, ultimately, mortality rates. Such factors have begun to be addressed in Scotland by the establishment of the Scottish Cancer Therapy Network (Aitken *et al.*, 1994).

### Conclusion

The Scottish Cancer Target, established in 1991, proposed that a reduction of 15% in mortality from cancer in the under-65s should be achieved between 1986 and 2000. On the basis of this analysis that target will be met. Mortality from cancer in those under 65 years of age between 1986 and 2000 in Scotland will fall by 17% in men and 25% in women. In contrast, however, it is projected that the number of new cancers diagnosed in this age group over the same time period will decrease by 6% in men but remain constant in women. We regret that the cancer target published for Scotland addresses only one aspect of cancer control, namely mortality. Without examination of the interplay between incidence and mortality it is impossible to obtain a sound understanding of the situation. Cancer control demands such an integrated approach.

**Acknowledgements**
The authors would like to express thanks to the General Register Office (Scotland) who gave permission for the use of individual cancer mortality records for this study. Thanks are also due to the staff in the regional cancer registries, staff based in the registration section of the Scottish Cancer Intelligence Unit and James Boyd for computing assistance. We gratefully acknowledge the cancer registries of Cracow, Denmark, England and Wales, South Netherlands and Tarragona, which are the sources of the comparative incidence and mortality data used in this paper. We are also grateful to the Health Monitoring Group of the Scottish Office and the grantholders of the Scottish Cancer Therapy Network for useful discussions on the paper.

### References

AITKEN REG, FARQUHAR W AND MOIR ATB. (1994). Cancer: advisory and co-ordinating bodies. *Health Bull.*, **52**, 47–50.

BARKER DJP. (1984). Time trends in cancer mortality in England and Wales. *Br. Med. J.*, **288,** 1325–1326.

BIRCH JM, MARSDEN HB, MORRIS JONES PH, PEARSON D AND BLAIR V. (1988). Improvements in survival from childhood cancer: results of a population based survey over 30 years. *Br. Med. J.*, **296,** 1372–1376.

BLACK RJ, SHARP L AND KENDRICK SW. (1993). *Trends in Cancer Survival in Scotland, 1968–90.* National Health Service in Scotland, Information & Statistics Division. ISD Publications: Edinburgh.

BLACK RJ, MACFARLANE G, MAISONNEUVE P AND BOYLE P. (1995). *Cancer Incidence and Mortality in Scotland, 1960–1989.* National Health Service in Scotland, Information & Statistics Division. ISD Publications: Edinburgh.

BOYLE P, KAYE SB AND ROBERTSON AG. (1987). Changes in testicular cancer in Scotland. *Eur. J. Cancer Clin. Oncol.*, **23,** 827–830.

BOYLE P, SOUKOP M, SCULLY C, ROBERTSON AG, BURNS MJ, GILLIS CR AND KAYE SB. (1988). Improving prognosis of Hodgkin's disease in Scotland. *Eur. J. Cancer Clin. Oncol.*, **24,** 229–234.

BOYLE P. (1989). Relative value of incidence and mortality data in cancer research. *Recent Results Cancer Res.*, **114,** 41–63.

BRESLOW NE. (1984). Extra-Poisson variation in log-linear models. *Appl. Stat.*, **33,** 38–44.

CLAYTON D AND SCHIFFLERS E. (1987). Models for temporal variation in cancer rates. I: Age-period and age-cohort models. *Stat. Med.*, **6,** 449–467.

EUROPEAN NETWORK OF CANCER REGISTRIES. (1995). *European Cancer Incidence and Mortality Database (EUROCIM) User Manual,* 2nd Edition. International Agency for Research on Cancer: Lyon.

GILLIS CR, HOLE DJ, STILL RM, DAVIS J AND KAYE SB. (1991). Medical audit, cancer registration and survival in ovarian cancer. *Lancet*, **337,** 611–612.

HARDING MJ, PAUL J, GILLIS CR AND KAYE SB. (1993). Management of malignant teratoma: does referral to a specialist unit matter? *Lancet*, **341,** 999–1002.

HAWKINS MM. (1989). Long term survival and cure after childhood cancer. *Arch. Dis. Child.*, **64,** 798–807.

HIGGINSON J, MUIR CS AND MUNOZ N. (1992). *Human Cancer: Epidemiology and Environmental Causes.* Cambridge University Press: Cambridge.

INFORMATION & STATISTICS DIVISION. (1994). *Scottish Health Statistics, 1994.* National Health Service in Scotland, Information & Statistics Division. ISD Publications: Edinburgh.

JUNOR EJ, HOLE DJ AND GILLIS CR. (1994). Management of ovarian cancer: referral to a multidisciplinary team matters. *Br. J. Cancer*, **70,** 363–370.

KARJALAINEN S. (1990). Geographical variation in cancer patient survival in Finland: chance, confounding or effect of early treatment? *J. Epidemiol. Comm. Health*, **44,** 210–214.

KARJALAINEN S AND PALVA I. (1989). Do treatment protocols improve end results? A study of survival of patients with multiple myeloma in Finland. *Br. Med. J.*, **299,** 1069–1072.

MCARDLE CS AND HOLE D. (1991). Impact of variability among surgeons on postoperative morbidity and mortality and ultimate survival. *Br. Med. J.*, **302,** 1501–1505.

MACGREGOR JE, CAMPBELL MK, MANN EMF AND SWANSON KY. (1994). Screening for cervical intraepithelial neoplasia in north east Scotland shows fall in incidence and mortality from invasive cancer with concomitant rise in preinvasive disease. *Br. Med. J.*, **308,** 1407–1411.

MACKIE RM AND HOLE D. (1992). Audit of public education campaign to encourage earlier detection of malignant melanoma. *Br. Med. J.*, **304,** 1012–1015.

MILLER BA, RIES LG, HANKEY BF, KOSARY CL, HARRAS F, DEVESA SS AND EDWARDS BK. (1993). *SEER Cancer Statistics Review 1973–1990.* NIH Publication No. 93–2789: Bethesda.

MUIR CS. (1993). Cancer registry in cancer control: An overview with a Scottish dimension. *Health Bull.* **51,** 208–229.

NATIONAL ALGORITHMS GROUP. (1985). *Generalised Linear Interactive Modelling Program (GLIM) Version 3.77.*

PETO J. (1989). Oral contraceptives and breast cancer: is the CASH study really negative? *Lancet*, **1,** 522.

PONTEN J, ADAMI H AND SPAREN P. (1991). Trends in cancer survival and mortality rates. *Med. Oncol. Tumour Pharm.*, **8,** 147–153.

REGISTRAR GENERAL SCOTLAND. (1981–90). *Annual Reports 1981–90.* HMSO: Edinburgh.

REGISTRAR GENERAL SCOTLAND. (1993). *Population Projections Scotland (1991 Based).* HMSO: Edinburgh.

ROBERTSON C AND BOYLE P. (1986). Age, period and cohort models: the use of individual records. *Stat. Med.*, **5,** 527–536.

SCOTTISH HOME AND HEALTH DEPARTMENT. (1991). *Health Education in Scotland: A National Policy Statement.* HMSO: Edinburgh.

SCOTTISH HOME AND HEALTH DEPARTMENT. (1992). *Scotland's Health: A Challenge to Us All.* HMSO: Edinburgh.

SCOTTISH HOME AND HEALTH DEPARTMENT. (1993). *The Scottish Diet.* Report of a Working Party to the Chief Medical Officer for Scotland. HMSO: Edinburgh.

SEGI M. (1960). *Cancer Mortality for Selected Sites in 24 Countries (1950–1957).* Tohoku University School of Medicine: Japan.

SHARP L, BLACK RJ, HARKNESS EF, FINLAYSON AR AND MUIR CS. (1993). *Cancer Registration Statistics, Scotland 1981–1990.* National Health Service in Scotland, Information & Statistics Division. ISD Publications: Edinburgh.

STILLER CA. (1994). Centralised treatment, entry to trials and survival. *Br. J. Cancer.*, **70,** 352–362.

STRONG JA. (1986). *The Cervical Cytology Service in Scotland.* Report by the Ad Hoc Group of the Histopathology Sub-committee of the Scientific Service Advisory Group chaired by Professor JA Strong. SHHD: Edinburgh.

TABAR L, FUGERBERG CJG, GAD A, BALDETORP L, HOLMBERG LH, GRÖNTOFT O, LJUNQVIST V, LUNDSTRÖM B, MÅNSON JL, EKLUND G, DAY NE AND PETTERSSON F. (1985). Reduction in mortality from breast cancer after mass screening with mammography. Randomised trial from the breast cancer screening working group of the Swedish National Board of Health and Welfare. *Lancet*, **1,** 829–832.

TOMATIS L. (1990). *Cancer Causes, Occurrence and Control.* International Agency for Research on Cancer Scientific Publications No. 100. IARC: Lyon.

UK NATIONAL CASE–CONTROL STUDY GROUP. (1989). Oral contraceptive use and breast cancer risk in young women. *Lancet*, **1,** 973–982.

WARNER J, BEATTIE C AND SHARP L. (1993). *Scottish Breast Screening Programme Report.* National Health Service in Scotland, Information & Statistics Division. ISD Publications: Edinburgh.

WORLD HEALTH ORGANIZATION. (1990). *Diet, Nutrition and the Prevention of Chronic Diseases.* Technical Report Series 797. WHO: Geneva.


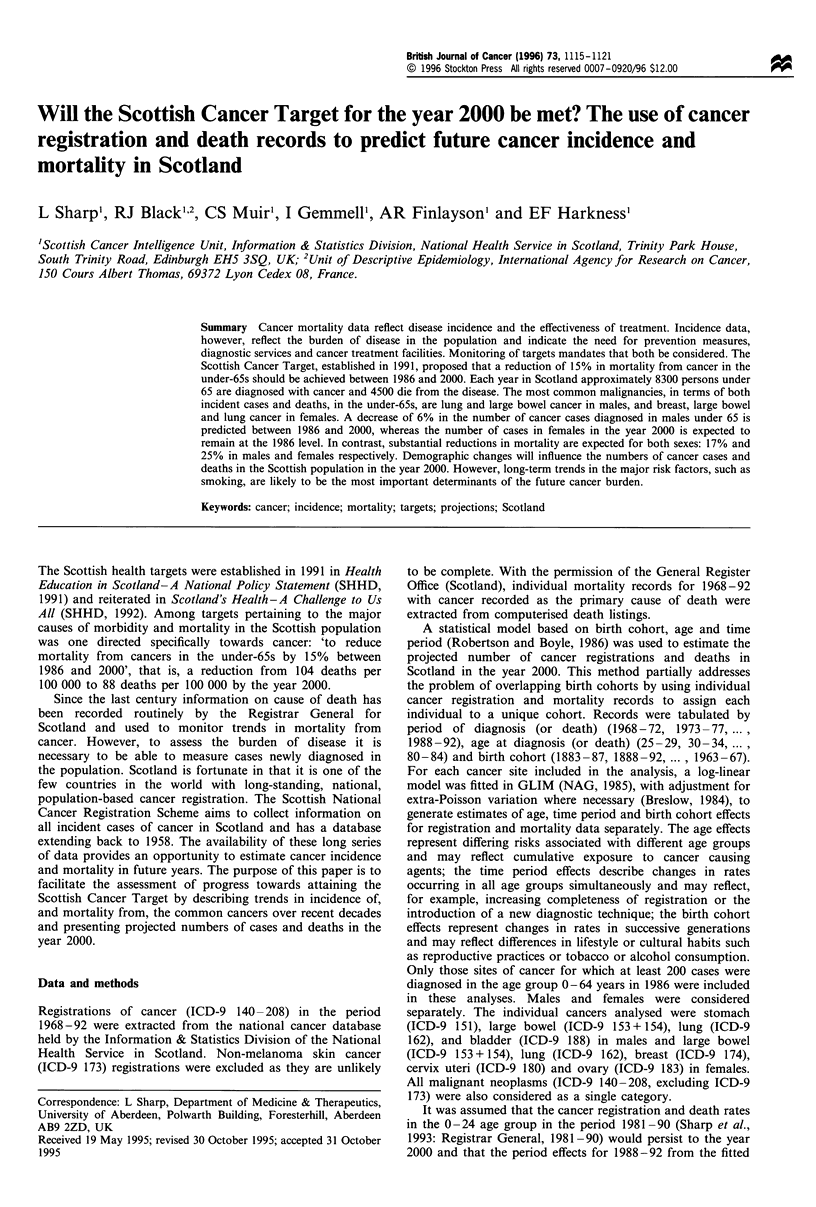

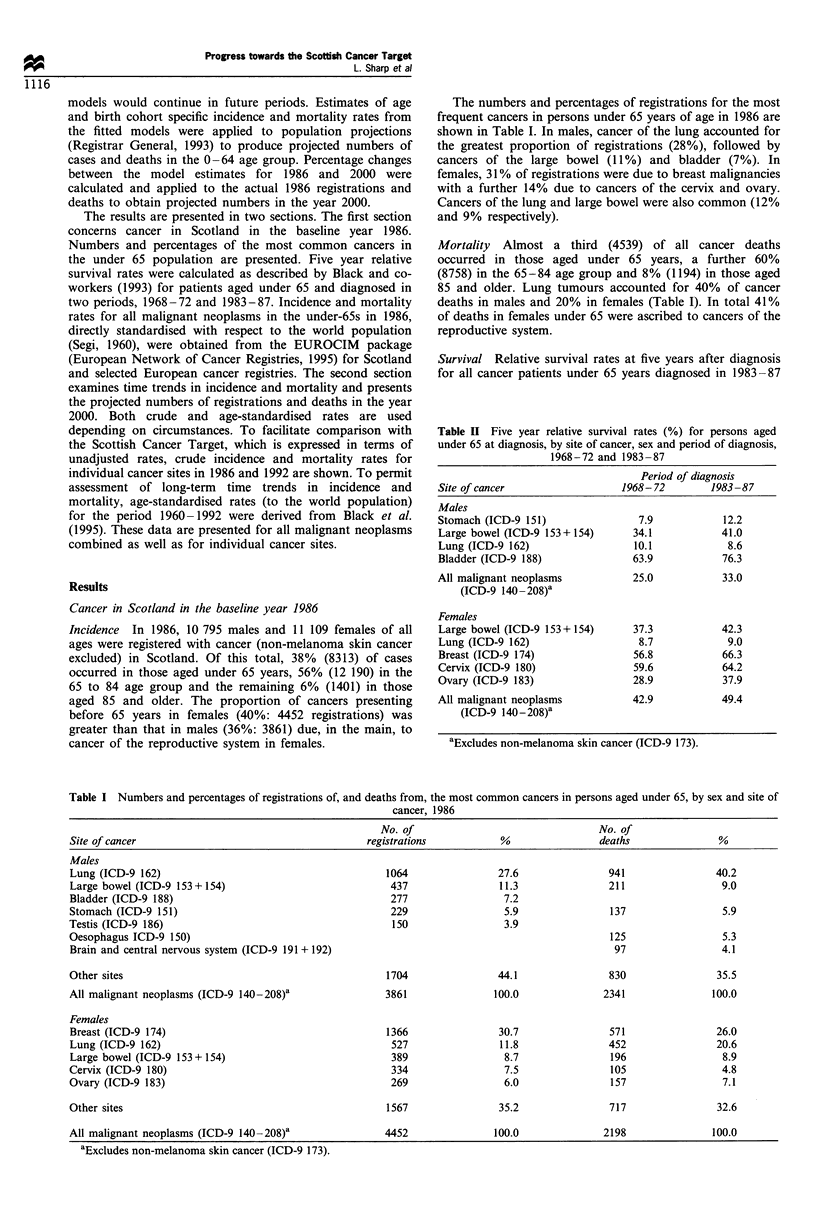

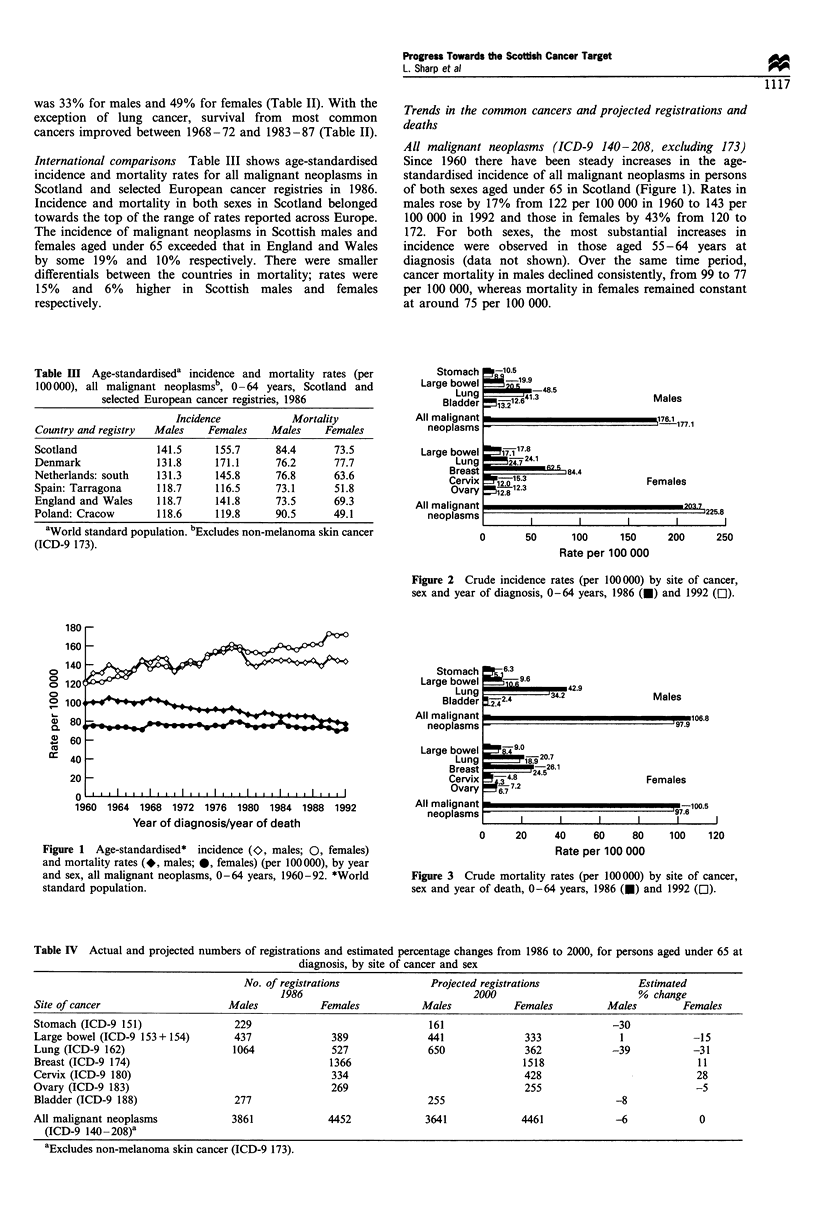

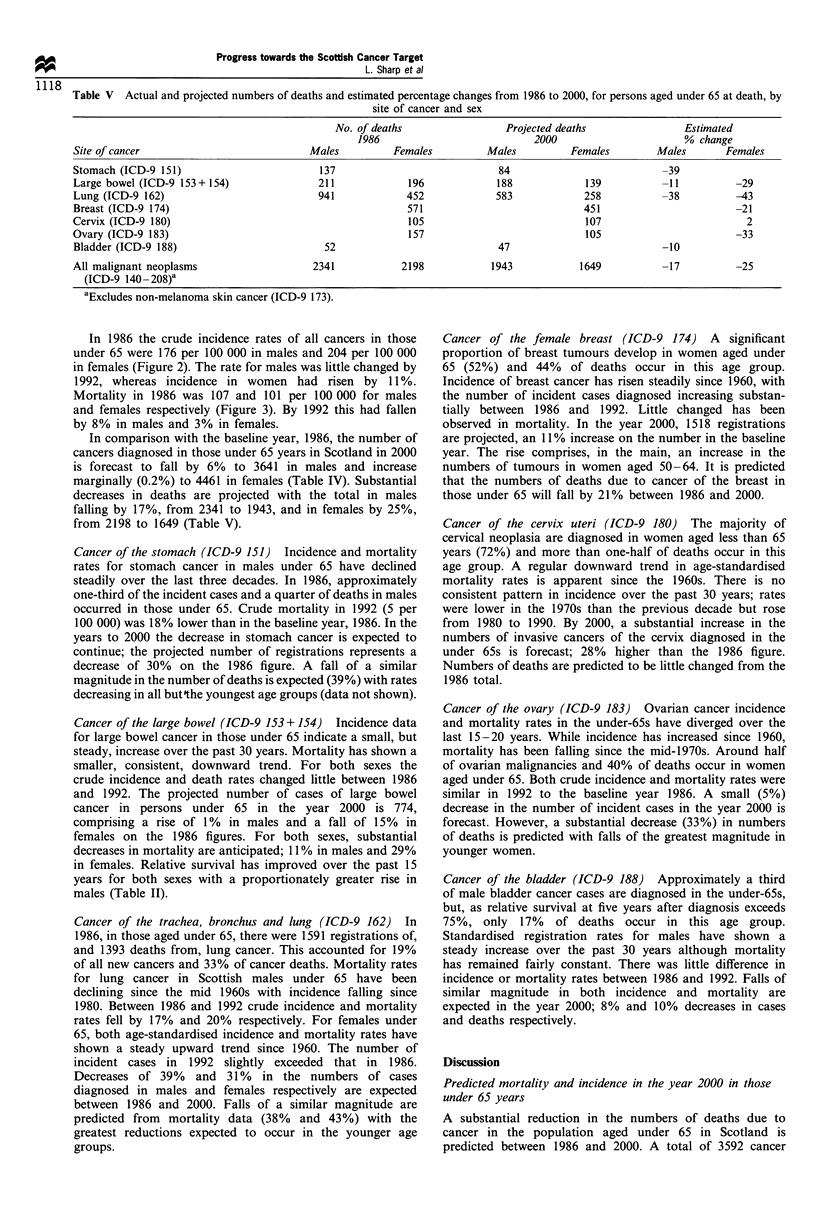

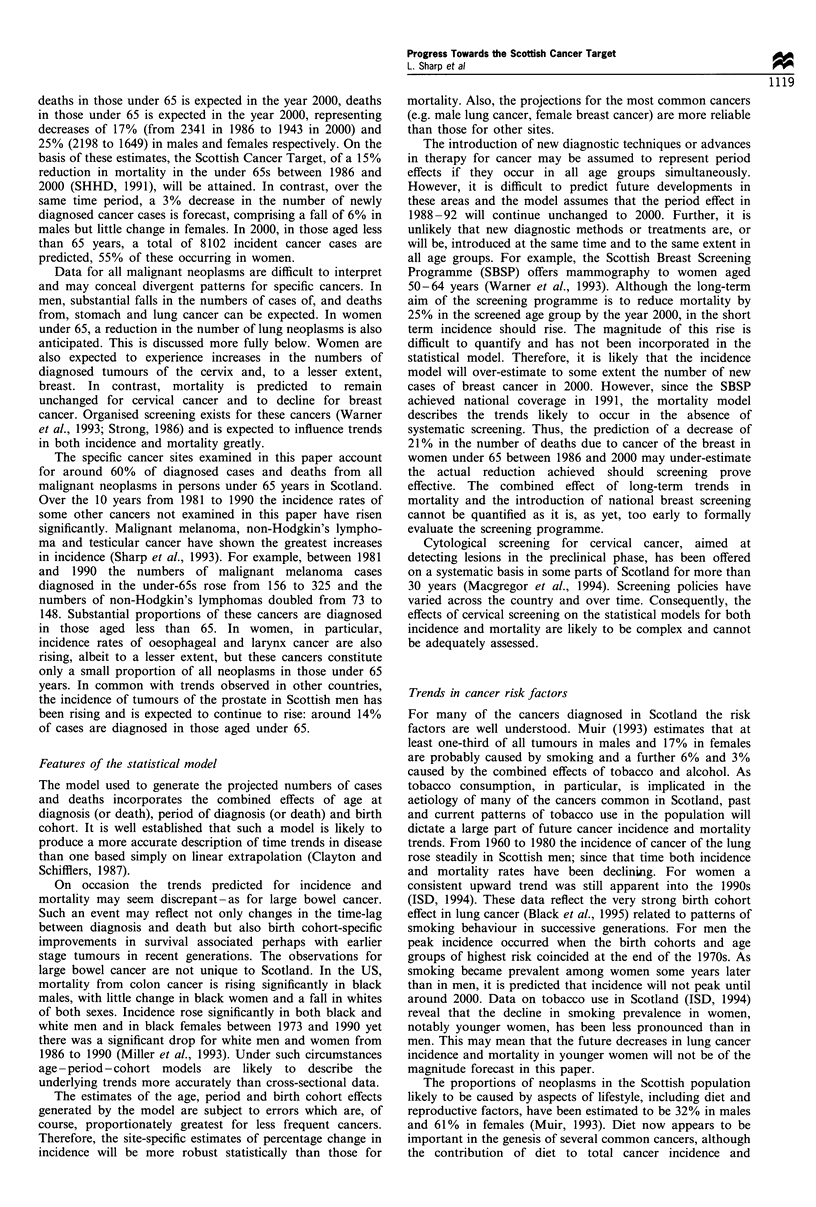

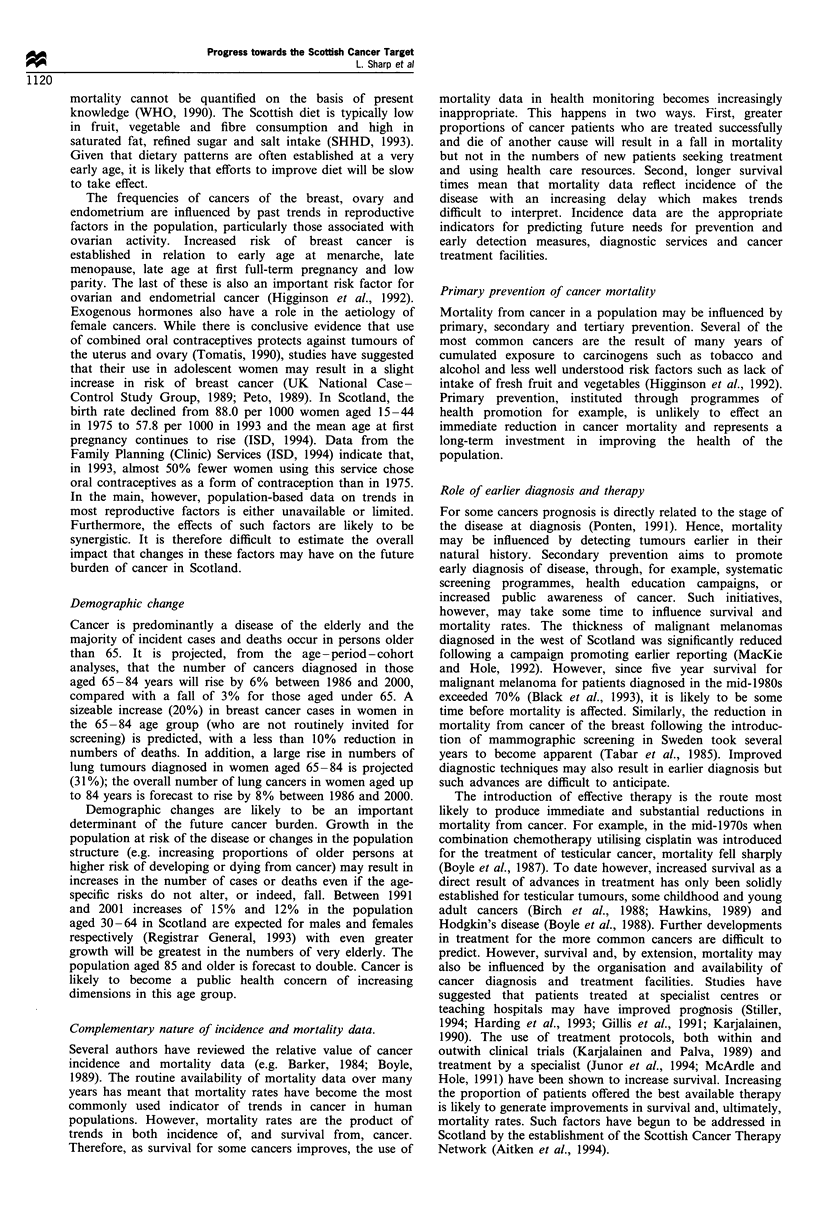

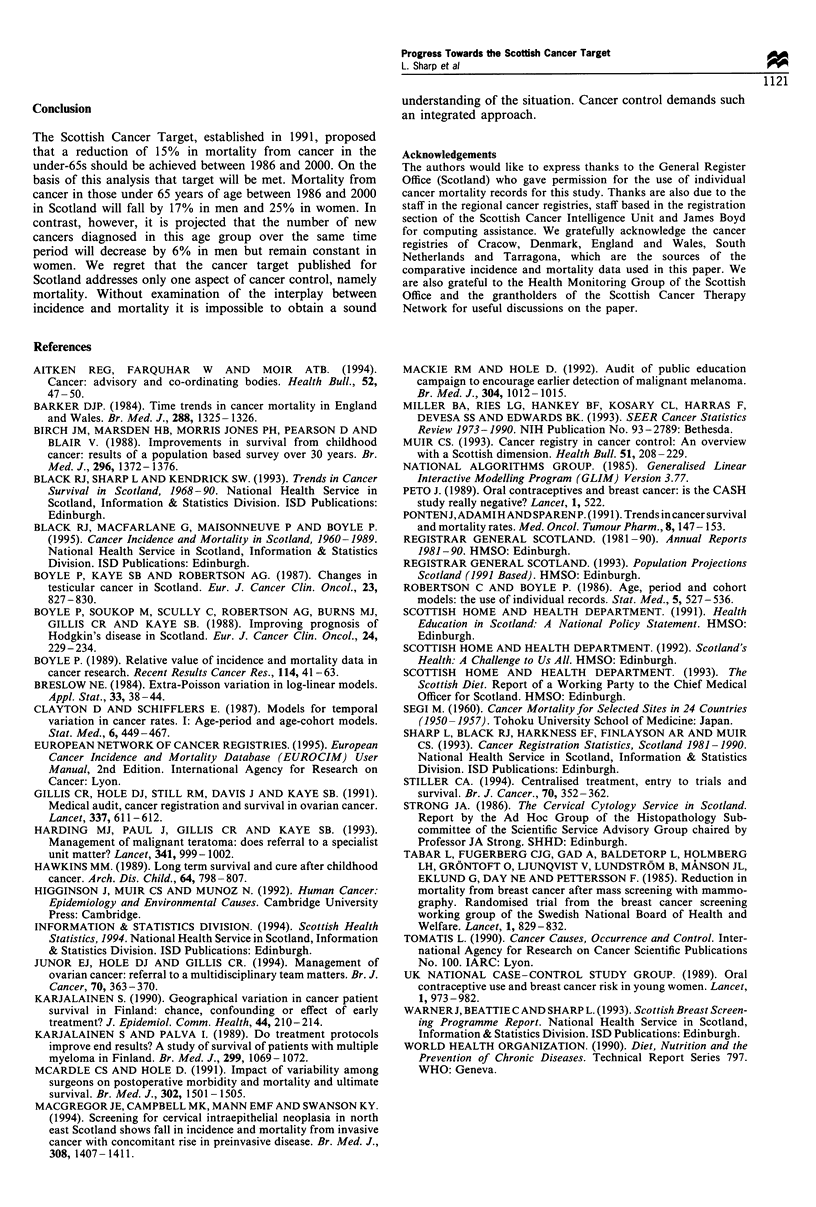


## References

[OCR_01039] Barker D. J. (1984). Time trends in cancer mortality in England and Wales.. Br Med J (Clin Res Ed).

[OCR_01063] Boyle P., Kaye S. B., Robertson A. G. (1987). Changes in testicular cancer in Scotland.. Eur J Cancer Clin Oncol.

[OCR_01066] Boyle P., Soukop M., Scully C., Robertson A. G., Burns H. J., Gillis C. R., Kaye S. B. (1988). Improving prognosis of Hodgkin's disease in Scotland.. Eur J Cancer Clin Oncol.

[OCR_01080] Clayton D., Schifflers E. (1987). Models for temporal variation in cancer rates. I: Age-period and age-cohort models.. Stat Med.

[OCR_01091] Gillis C. R., Hole D. J., Still R. M., Davis J., Kaye S. B. (1991). Medical audit, cancer registration, and survival in ovarian cancer.. Lancet.

[OCR_01096] Harding M. J., Paul J., Gillis C. R., Kaye S. B. (1993). Management of malignant teratoma: does referral to a specialist unit matter?. Lancet.

[OCR_01101] Hawkins M. M. (1989). Long term survival and cure after childhood cancer.. Arch Dis Child.

[OCR_01115] Junor E. J., Hole D. J., Gillis C. R. (1994). Management of ovarian cancer: referral to a multidisciplinary team matters.. Br J Cancer.

[OCR_01120] Karjalainen S. (1990). Geographical variation in cancer patient survival in Finland: chance, confounding, or effect of treatment?. J Epidemiol Community Health.

[OCR_01127] Karjalainen S., Palva I. (1989). Do treatment protocols improve end results? A study of survival of patients with multiple myeloma in Finland.. BMJ.

[OCR_00106] Lung M. L., Lam W. P., Chan K. H., Li S., Sham J., Choy D. (1992). Direct detection of Epstein-Barr virus in peripheral blood and comparison of Epstein-Barr virus genotypes present in direct specimens and lymphoblastoid cell lines established from nasopharyngeal carcinoma patients and healthy carriers in Hong Kong.. Int J Cancer.

[OCR_01137] Macgregor J. E., Campbell M. K., Mann E. M., Swanson K. Y. (1994). Screening for cervical intraepithelial neoplasia in north east Scotland shows fall in incidence and mortality from invasive cancer with concomitant rise in preinvasive disease.. BMJ.

[OCR_01132] McArdle C. S., Hole D. (1991). Impact of variability among surgeons on postoperative morbidity and mortality and ultimate survival.. BMJ.

[OCR_01178] Robertson C., Boyle P. (1986). Age, period and cohort models: the use of individual records.. Stat Med.

